# Loss of Brcc3 in Zebrafish Embryos Increases Their Susceptibility to DNA Damage Stress

**DOI:** 10.3390/ijms252212108

**Published:** 2024-11-11

**Authors:** Zhengyang Wang, Caixia Wang, Yanpeng Zhai, Yan Bai, Hongying Wang, Xiaozhi Rong

**Affiliations:** 1 Key Laboratory of Marine Drugs (Ocean University of China), Chinese Ministry of Education, and School of Medicine and Pharmacy, Ocean University of China, 5 Yushan Road, Qingdao 266003, China; 2 Laboratory for Marine Drugs and Bioproducts, Qingdao Marine Science and Technology Center, Qingdao 266237, China; 3 Hubei Provincial Key Laboratory for Protection and Application of Special Plants in Wuling Area of China, Key Laboratory of State Ethnic Affairs Commission for Biological Technology, College of Life Sciences, South-Central Minzu University, Wuhan 430074, China

**Keywords:** ATM, Brcc3, CRISPR/Cas9, zebrafish, DNA damage, p53, UV, ETO

## Abstract

DNA double-strand breaks (DSBs) represent one of the most severe forms of genetic damage in organisms, yet vertebrate models capable of monitoring DSBs in real-time remain scarce. BRCA1/BRCA2-containing complex subunit 3 (BRCC3), also known as BRCC36, functions within various multiprotein complexes to mediate diverse biological processes. However, the physiological role of BRCC3 in vertebrates, as well as the underlying mechanisms that govern its activity, are not well understood. To explore these questions, we generated *brcc3*-knockout zebrafish using CRISPR/Cas9 gene-editing technology. While *brcc3* mutant zebrafish appear phenotypically normal and remain fertile, they exhibit significantly increased rates of mortality and deformity following exposure to DNA damage. Furthermore, embryos lacking Brcc3 display heightened p53 signaling, elevated γ-H2AX levels, and increased apoptosis in response to DNA-damaging agents such as ultraviolet (UV) light and Etoposide (ETO). Notably, genetic inactivation of p53 or pharmacological inhibition of Ataxia-telangiectasia mutated (ATM) activity rescues the hypersensitivity to UV and ETO observed in Brcc3-deficient embryos. These findings suggest that Brcc3 plays a critical role in DNA damage response (DDR), promoting cell survival during embryogenesis. Additionally, *brcc3*-null mutant zebrafish offer a promising vertebrate model for real-time monitoring of DSBs.

## 1. Introduction

DNA, as a carrier of genetic information, is essential for maintaining its structural integrity and stability during replication. However, DNA continuously suffers from endogenous and exogenous damage [1]. In vertebrate cells, the DNA damage response (DDR) is regulated by three kinds of apical kinases: ATM, ATM- and Rad3-related (ATR) and DNA-dependent protein kinase (DNA-PK), which assist in the detection of DNA damage and facilitate repair processes [2]. DNA double-strand breaks (DSBs) can be repaired by homologous recombination (HR) and non-homologous end joining (NHEJ), and other repair methods, such as base-excision repair (BER) and nucleotide excision repair (NER), can repair single-strand breaks (SSBs) [3,4]. Additionally, cells also activate the p53 signaling pathway for self-elimination in response to severe DNA damage [1,5,6].

After DSBs, a protein complex, BRCA1-A, targets DNA damage sites and/or replication forks through the signaling cascade of DDR and participates in HR-dependent repair [4,7]. The BRCA1-A complex consists of five stoichiometric components, including Abraxas1, RAP80, BRCC45, BRCC3, and MERIT40 [7,8]. As a HR repair protein, BRCA1 mutations are often associated with a high incidence of tumors [4,9,10]. However, the embryonic lethality of BRCA1 homozygous mutant mice limits its studies in vivo [11]. Recent research has shown that MERIT40 mutant mice were sensitive to the DNA interstrand cross-links inducer mitomycin C, and significantly delayed the replication fork progression. While they are not affected by whole-body irradiation [12]. Remarkably, the MERIT40 mutant mice were viable and exhibited a normal phenotype in the absence of exogenous genotoxic stress [12]. These results suggest that we can alternatively utilize the components of the BRCA1-A complex to develop viable vertebrate models that are sensitive to DNA damage and to explore the physiological roles of these components for the in vivo evaluation of specific selectivity of chemical compounds targeting these for potentially therapeutic application.

In the complex of BRCA1-A, BRCC3 is the only enzymatically active component [8,13,14,15]. It contains an N-terminal JAMM/MPN+ domain which specifically cleaves the K63-linked ubiquitin chains [8,16,17,18]. K63-linked ubiquitination of H2AX can activate the DDR-induced ubiquitination cascade at DNA damage sites, and these K63-linked ubiquitination can be removed by BRCC3 specifically [4,19,20,21]. These results suggest that BRCC3 may be pivotal in DNA damage repair. *Brcc3*-knockout mice are fertile and have no gross phenotypic abnormalities [7]. However, due to mouse embryos developing in utero with long gestation period, it is not convenient for DNA damage sensitivity evaluation. Therefore, the in vivo biological function of BRCC3, especially in relation to DDR, has not been well investigated.

In the field of DDR studies, the cultured cell models are convenient and controllable, but lack complexity. In comparison to cultured cells, the zebrafish embryos provide a more advantageous platform for in vivo studies of the physiological function of DDR related genes. For example, zebrafish embryos are suitable for sampling and performing in vivo experiments owing to their optical transparency, external and rapid development, as well as large clutch size [22,23]. Given the BRCC3 is evolutionarily conserved between zebrafish and humans, we therefore generated *brcc3*-null zebrafish, which effectively process and visualize the phenotype of the DNA damage response in vivo.

We found the *brcc3* mutant zebrafish are phenotypically normal and fertile, although the *brcc3* mutant embryos exhibit relatively elevated p53 signaling pathway. Interestingly, after induction of double-strand breaks (DSBs) in zebrafish embryos using UV or ETO, the mortality and deformity rates of *brcc3* homozygous mutant zebrafish were significantly higher than those of wild-type. In addition, genetic inactivation of p53 or pharmacological inhibition of ATM activity counteracts the Brcc3 depletion-induced sensitive responsiveness to the UV or ETO treatment. These results indicate that *brcc3* mutants can be used to detect DNA double-strand break toxicity induced either by drugs or by environmental radiation. Importantly, the *brcc3* mutants also hold potential as experimental models for anticancer drug screening.

## 2. Results

### 2.1. Loss of Brcc3 Is Tolerated in Zebrafish

**Figure 1:**
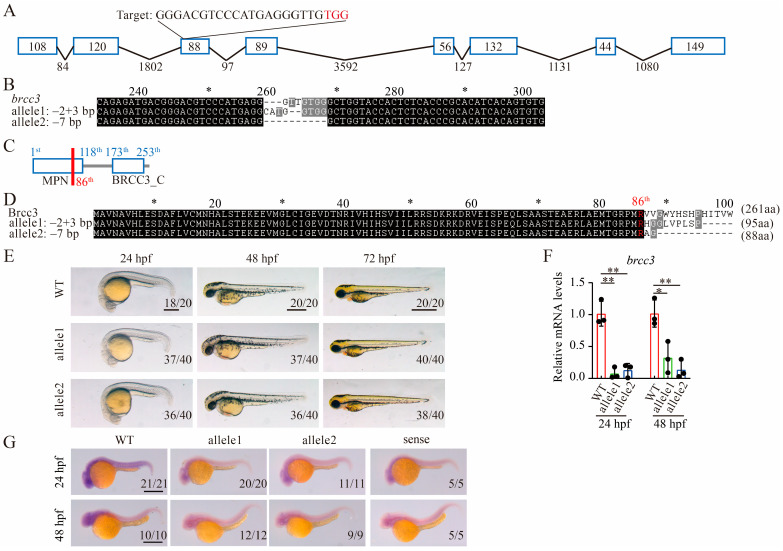
Generation of *brcc3* knockout zebrafish by using CRISPR/Cas9. (**A**) Schematic diagram of zebrafish *brcc3* gene and CRISPR/Cas9 target site. The blue box represents the exon, and the black line represents the intron. CRISPR/Cas9 gRNA target site locates in the third exon and the target sequence is indicated at the top of the image. Protospacer adjacent motif (PAM) region is shown in red. (**B**) The generated zebrafish *brcc3*-null alleles. (**C**) Schematic diagram of zebrafish Brcc3 domains. The red line indicates the initial abnormal translation site of the mutant *brcc3* alleles. (**D**) The predicted protein sequences of WT and mutant *brcc3* alleles are shown. (**E**) Representative images of WT, MZ*brcc3*^−2+3/−2+3^, and MZ*brcc3*^−7/−7^ mutant embryos at 24, 48, and 72 hpf. Lateral views with anterior to the left. Scale bar = 500 μm. (**F**) The relative mRNA levels of *brcc3* in WT and mutant embryos at 24 and 48 hpf, as indicated by qRT-PCR analysis. The results are from three independent replicates. Values are represented as means ± s.d. * *p* < 0.05; ** *p* < 0.01 (unpaired two-tailed Student’s *t*-test). (**G**) Expression of *brcc3* in WT and *brcc3* mutant embryos at 24 and 48 hpf. The sense probe of *brcc3* was used as a control. Scale bar = 500 μm. The proportion of embryos with the indicated phenotypes is shown in the bottom right corner of each panel.

To investigate the role of Brcc3 in zebrafish, we used CRISPR/Cas9 to create two *brcc3* knockout mutant lines, −2 bp + 3 bp and −7 bp, by targeting the third exon (Figure 1A,B). The zebrafish Brcc3 protein contains two domains: the MPN domain (amino acids 1–118) and the BRCC3 carbon-terminal domain (amino acids 173–253) (Figure 1C). Both null mutants exhibited frame-shift mutations, resulting in a truncated Brcc3 protein (predicted to be 86 amino acids long) (Figure 1C,D). The adult *brcc3* zygotic mutant fish were obtained. Then, the adult female and male homozygous fish were crossed to generate maternal zygotic mutant embryos to eliminate maternally derived mRNA or protein for phenotypic analysis. No morphological differences were observed between wild-type and mutant embryos at various stages (24, 48, and 72 hpf), or in adult fish (Figure 1E). To verify the alleles are null alleles, we measured the mRNA levels of *brcc3* in WT and mutant embryos at 24 and 48 hpf by quantitative real-time RT-PCR (qRT-PCR) and whole-mount in situ hybridization analysis, respectively. Indeed, the transcripts of *brcc3* were greatly decreased in *brcc3* mutants at 24 and 48 hpf (Figure 1F,G). This might be a result of nonsense-mediated mRNA decay. Taken together, we concluded that depletion of Brcc3 is tolerated in zebrafish.

### 2.2. Depletion of Brcc3 in Zebrafish Embryos Alleviates the Resistance to UV Radiation

**Figure 2:**
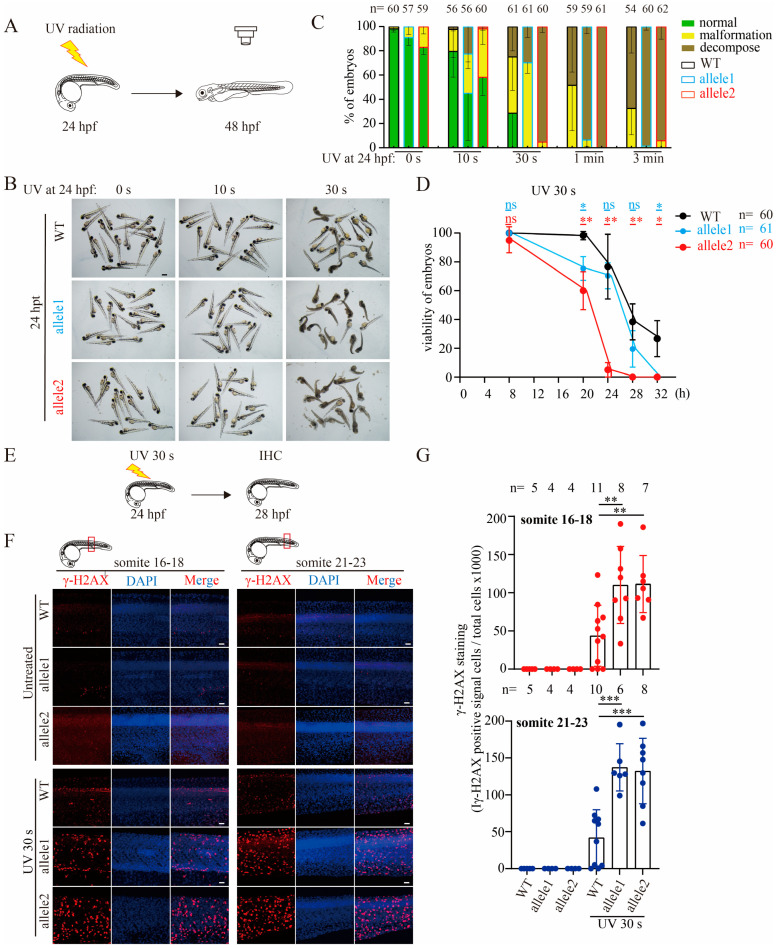
Depletion of Brcc3 in zebrafish embryos alleviates the resistance to UV radiation and increases the sensitivity to UV radiation-induced DNA damage. (**A**) Schematic diagram of zebrafish embryos radiated with UV. Embryos were radiated for different times of duration at 24 hpf and then raised to 48 hpf. (**B**) Representative images of WT and mutant embryos at 48 hpf after UV radiation. WT and mutant embryos at 24 hpf were treated with UV for 0 s, 10 s, and 30 s, and then raised to 48 hpf. hpt, hours-post treatment; Scale bar = 1 mm. (**C**) Quantitative results from images as shown in (**B**). WT and mutant embryos at 48 hpf in B were categorized into normal, malformed, and decomposed classes, respectively. Results are from three independent replicates. The total number of embryos in each group is shown above the column. (**D**) Survival curve of WT and mutant embryos after 30 s UV radiation. WT and mutant embryos at 24 hpf were treated with UV for 30 s and viability was continuously recorded at the indicated timepoint from 8 to 32 h after treatment. The total number of embryos in each group is shown in the upper right corner of the column. Values are represented as means ± s.d. ns, not significant; * *p* < 0.05; ** *p* < 0.01 (unpaired two-tailed Student’s *t*-test). (**E**) Schematic diagram of UV radiation experiments for immunohistochemical staining analysis. (**F**) Representative confocal images of WT and *brcc3* mutant embryos at 28 hpf which were immunostained with an anti-γ-H2AX antibody after UV radiation. Nuclei were counterstained with DAPI (blue). Somite 16 to 18 and 21 to 23 were selected for confocal imaging analysis, respectively. Scale bar = 10 μm. (**G**) Quantitative results from images as shown in (**F**). The γ-H2AX signal was represented by the γ-H2AX positive signal cells/total cells × 1000. Results are from three independent replicates. Values are represented as means ± s.d. ** *p* < 0.01; *** *p* < 0.001 (one-way ANOVA followed by Tukey’s post hoc test).

Previous studies reported that BRCC3 is involved in DNA damage repair [7,24]. UV irradiation leads to the formation of pyrimidine dimers, which NER-mediated repair is required, and clustered single-strand breaks formed during this process cause DSBs, eventually resulting in cell apoptosis [25,26]. To assess the response of *brcc3* mutants on UV irradiation-induced DSB, WT and mutant zebrafish embryos at 24 hpf were subjected to UV irradiation for various durations (0 s, 10 s, 30 s, 1 min, and 3 min) and then observed at 48 hpf (Figure 2A). We noticed that malformation and mortality rates of both WT and mutant embryos at 48 hpf were increased in a duration-dependent manner (Figure 2B–D). However, the malformation and mortality rates of *brcc3* mutant embryos were significantly higher than those of WT embryos (Figure 2B–D). These results suggested that zebrafish *brcc3* mutants are hypersensitive to UV radiation.

H2AX is a variant of histone H2A, plays a critical role in the DNA damage response [27,28]. Upon the occurrence of DSBs, kinases such as ATM and ATR swiftly phosphorylate serine 139 of H2AX at the DNA damage site, resulting in the formation of γ-H2AX [29]. Hence, γ-H2AX is considered as an indicator of DNA damage. The increased rates of malformation and mortality in *brcc3* mutant embryos suggested that depletion of Brcc3 in zebrafish embryos likely sensitized their response to UV radiation-induced DNA damage. To test this, we used an anti-γ-H2AX antibody to immunostain UV-treated *brcc3* mutant embryos at 28 hpf (Figure 2E). The levels of γ-H2AX in wild-type and mutant embryos exhibited no or little difference (Figure 2F,G). In contrast, after UV treatment, the levels of γ-H2AX in *brcc3* mutant were increased dramatically (Figure 2F,G). These results suggested that depletion of Brcc3 in zebrafish embryos leads to an increase in UV radiation-induced DNA damage.

### 2.3. Depletion of Brcc3 in Zebrafish Embryos Enhances Its Sensitivity to UV Radiation-Induced p53 Signaling; And Inactivation of p53 Strengthens the Resistance of brcc3 Mutant Embryos to UV Radiation

**Figure 3:**
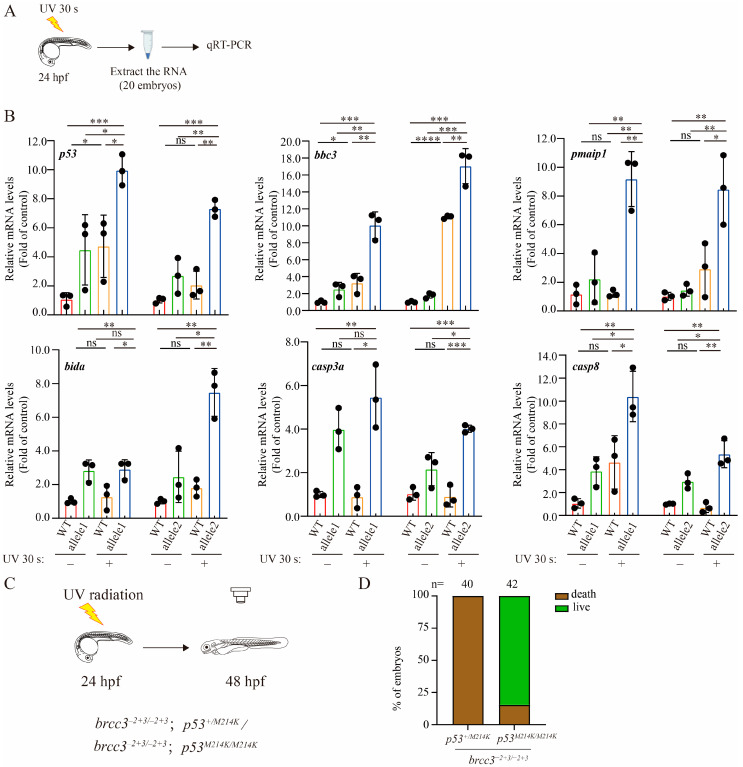
Depletion of Brcc3 in zebrafish embryos amplifies the up-regulatory response of p53 signaling to UV radiation. (**A**) Schematic diagram of UV radiation experiments for qRT-PCR analysis. (**B**) The relative mRNA levels of *p53* and its target genes in WT and mutant embryos with or without UV radiation are accessed by qRT-PCR. Results are from three independent replicates. Values are represented as means ± s.d. ns, not significant; * *p* < 0.05; ** *p* < 0.01; *** *p* < 0.001; **** *p* < 0.0001 (one-way ANOVA followed by Tukey’s post hoc test). (**C**) Schematic diagram of UV radiation experimental design to access the p53 action after Brcc3 depletion. (**D**) Survival rate of *brcc3* mutant embryos at 48 hpf in the *p53*^+/*M214K*^ or *p53^M214K/M214K^* genetic background. The *brcc3*^−2+3bp/−2+3bp^; *p53^+/M214K^* and *brcc3*^−2+3bp/−2+3bp^; *p53^M214K/M214K^* were crossed, and the offsprings at 24 hpf were subjected to UV radiation for 30 s. The survival embryos at 48 hpf were subjected to genotyping analysis. Values are represented as means ± s.d. The total number of embryos is shown on each column.

The UV radiation-induced malformation and mortality rates of both WT and mutant embryos were increased in a duration-dependent manner. Additionally, previous studies have shown that UV radiation induces p53 signaling pathway [30,31]. We therefore examined the transcriptional levels of *p53* and its downstream target genes, as well as two apoptotic genes *casp3a* and *casp8*, in UV radiation-treated zebrafish embryos. The WT or mutant zebrafish embryos with or without UV irradiation were subjected to quantitative RT-PCR analysis (Figure 3A). Depletion of Brcc3 resulted in little to slight induction of the mRNA levels of *p53* and its direct target genes *bbc3*, *pmaip1*, and *bida* as well as *casp3a* and *casp8* (Figure 3B). After UV radiation, however, the mRNA levels of these genes were synergistically increased in *brcc3* mutant embryos compared with those in WT embryos (Figure 3B). If activation of p53 signaling pathway is a major contribution to increased mortality rates of *brcc3* mutant embryos after UV irradiation, genetic inactivation of p53 would be expected to relieve this effect. Indeed, genetic inactivation of p53 increased the viability of *brcc3* mutant embryos after UV irradiation (Figure 3C,D). Taken together, the above results suggested that the dramatically increased mortality in *brcc3* mutant embryos following UV irradiation is likely due to Brcc3 depletion-induced hyperactivation of the p53 signaling pathway.

### 2.4. Depletion of Brcc3 in Zebrafish Embryo Enhances Its Sensitivity to ETO Treatment and Induction of p53 Signaling

**Figure 4:**
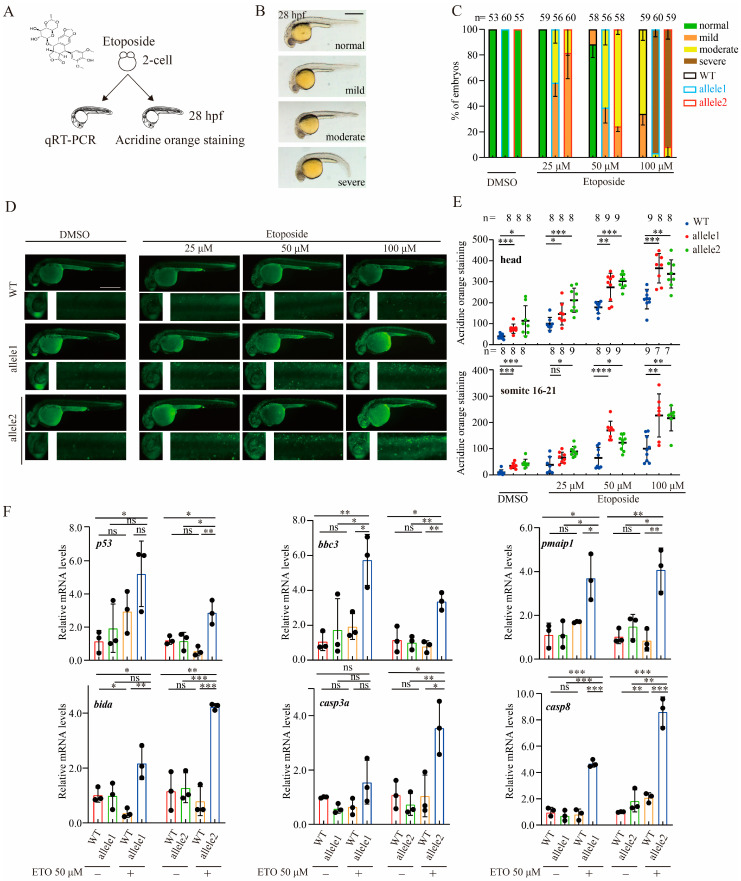
Brcc3-depleted zebrafish embryos exhibit increased sensitivity to ETO treatment. (**A**) Schematic diagram of ETO treatment of WT and *brcc3* mutant embryos for immunohistochemical staining and qRT-PCR analysis. (**B**) The various degrees of apoptosis of embryos after treatment with ETO. Scale bar = 500 μm. (**C**) Quantitative results from images as shown in (**B**). WT and *brcc3* mutant embryos at 28 hpf in (**B**) were categorized into each indicated group. Results are from three independent replicates. Values are represented as means ± s.d. The total number of embryos is shown at the top of each column. (**D**) Acridine orange staining of WT and *brcc3* mutant embryos at 28 hpf after ETO treatment with various doses. Scale bar = 500 μm. (**E**) Quantitative results from images of head (upper panel) or 16–21 somite (lower panel) region as shown in (**D**). The total number of embryos is shown on each column. Values are represented as means ± s.d. ns, not significant; * *p* < 0.05; ** *p* < 0.01; *** *p* < 0.001; **** *p* < 0.0001 (one-way ANOVA followed by Tukey’s post hoc test). (**F**) The relative mRNA levels of *p53* and indicated genes in WT and *brcc3* mutant embryos at 48 hpf with/without ETO treatment. Results are from three independent replicates. Values are represented as means ± s.d. ns, not significant; * *p* < 0.05; ** *p* < 0.01; *** *p* < 0.001 (one-way ANOVA followed by Tukey’s post hoc test).

The zebrafish *brcc3* mutant embryos appeared to be hypersensitive to UV-induced DNA damage. To further confirm that Brcc3-depleted zebrafish embryos are hypersensitive to DNA damage, we also used a chemical compound ETO, an inhibitor of topoisomerase II, which can induce DSBs by trapping topoisomerase II behind replication forks [32]. WT and *brcc3* mutant embryos were exposed to ETO from the 2-cell stage to 28 hpf to induce DSBs in vivo (Figure 4A). At 28 hpf, both WT and mutant embryos treated with ETO exhibited varying degrees of apoptosis (Figure 4B). The phenotypes of ETO-treated WT and mutant embryos were categorized as normal, mild, moderate, and severe based on the extent of apoptosis (Figure 4B). Notably, in the ETO-treated mutant group, the severity of the apoptotic phenotype increased in a concentration-dependent manner, showing a more pronounced effect compared to WT embryos (Figure 4C). Acridine orange (AO) can be used to label apoptotic cells through its selective permeability to the cell membrane and its different fluorescence colors when bound to DNA/RNA. To corroborate these findings, we performed AO staining to quantify apoptotic cells. Consistent with earlier observations, Brcc3-deficient embryos showed significantly elevated levels of apoptosis compared to WT embryos following ETO treatment (Figure 4D). Images of the zebrafish embryos were captured, specifically focusing on the head and somite regions 16–21, and apoptotic cells were manually counted. Apoptosis was observed to increase in a dose-dependent manner in both WT and mutant embryos, with significantly higher counts in the *brcc3* mutants at all ETO concentrations (Figure 4E). Previous studies have shown that ETO can induce phosphorylation of p53 and increase the level of total p53 [33]. We speculated that the p53 signaling was overactivated in *brcc3* mutant embryos when embryos were treated with ETO. Hence, WT and *brcc3* mutant embryos at 28 hpf with or without ETO treatment were used to determine the transcriptional levels of *p53* and its downstream target genes *bbc3*, *pmaip1*, and *bida*, as well as the apoptotic genes *casp3a* and *casp8*, by qRT-PCR analysis, respectively (Figure 4F). Similar to the results of UV radiation-treated WT and *brcc3* mutant embryos, treatment with ETO also led to a robust increase in *p53* and its downstream target genes *bbc3*, *pamip1*, and *bida1*, as well as the apoptotic genes *casp3a* and *casp8* in *brcc3* mutant embryos but not WT embryos (Figure 4F). In summary, these results suggest that the loss of Brcc3 in zebrafish embryos increases their sensitivity to ETO treatment, likely through overactivation of the p53 signaling pathway.

### 2.5. Inhibition of ATM Activation Mitigates the Enhanced UV Radiation-Induced Effects After Brcc3 Depletion

**Figure 5:**
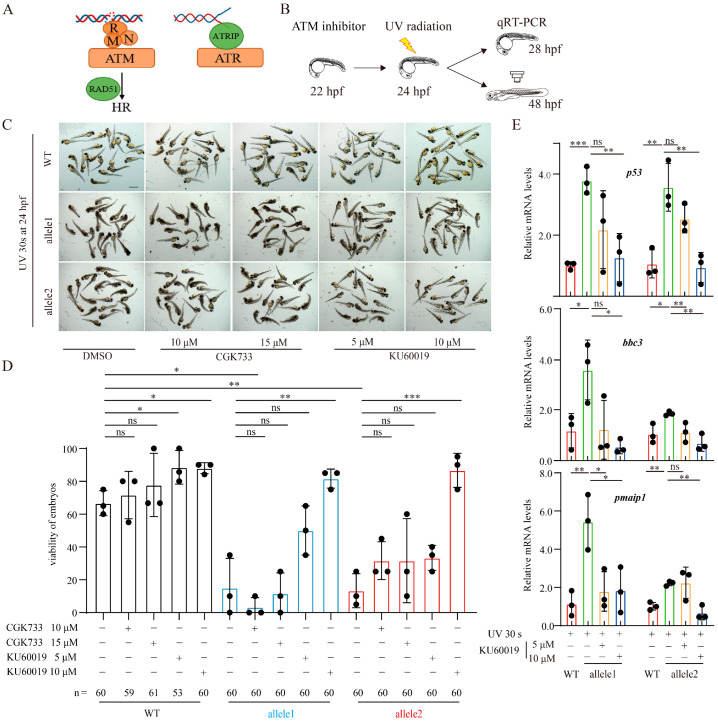
Pharmacological inhibition of ATM activation mitigates the enhanced UV radiation-induced effects after Brcc3 depletion. (**A**) Model for ATM or ATR in Response to DNA Damage. (**B**) Schematic diagram of qRT-PCR or phenotypic analysis of embryos after UV radiation with/without ATM inhibitors. WT and *brcc3* mutant embryos at 22 hpf with/without ATM inhibitors treated were raised to 24 hpf, treated with UV radiation for 30 s, and then subjected for qRT-PCR or phenotypic analysis at indicated timepoint. (**C**) Representative images of UV radiation-treated WT and *brcc3* mutant embryos at 48 hpf in addition of different doses of ATM/ATR inhibitors. Scale bar = 1 mm. (**D**) The viability of WT and *brcc3* mutant embryos in (**B**). Values are represented as means ± s.d. ns, not significant; * *p* < 0.05; ** *p* < 0.01; *** *p* < 0.001. (one-way ANOVA followed by Tukey’s post hoc test). (**E**) The relative mRNA levels of *p53* and its target genes in UV radiation-treated WT and *brcc3* mutant embryos at 28 hpf with/without KU60019 inhibitor. Results are from three independent replicates. Values are represented as means ± s.d. ns, not significant; * *p* < 0.05; ** *p* < 0.01; *** *p* < 0.001. (one-way ANOVA followed by Tukey’s post hoc test).

Previous studies have indicated that UV radiation-induced DNA damage activates the ATM and ATR kinases in response to DSBs or DNA replication stress [2,30,34]. In addition, the deficiency of BRCC3 DUB was shown to result in ionizing radiation (IR) hypersensitivity and enhanced IR-induced cell apoptosis [7,19,24]. ATM or ATR is the primary kinase responsible to DNA damage, and they can be recruited to DSBs or ssDNA in the presence of MRE11-RAD50-NBS1 (MRN) complex or ATRIP, respectively (Figure 5A) [2]. Moreover, the Brcc3-depleted zebrafish are hypersensitive responsiveness to UV radiation-induced DNA damage, we utilized the ATM and ATM/ATR chemical inhibitors, KU60019 and CGK733, to investigate whether inhibition of ATM or ATM/ATR could alleviate the effects in zebrafish embryos after depletion of Brcc3. As shown in Figure 5B, the UV-irradiated WT or *brcc3* mutant embryos were treated with different doses of KU60019 or CGK733, respectively. Then the inhibitor-treated embryos were raised to appropriately developmental stage and subjected to phenotypic and qRT-PCR analysis, respectively. Addition of KU60019 while not CGK733 significantly restored normal development of UV radiation-induced increase in the malformation and mortality of *brcc3* mutant embryos (Figure 5C,D). Since the p53 signaling pathway functions downstream of ATM, we therefore examined the effect of the addition of KU60019 on the UV radiation-induced enhancement of mRNA levels of *p53* and its target genes, *bbc3* and *pmaip1*, in *brcc3* mutant embryos. Indeed, treatment of KU60019 restored the UV radiation-induced increased mRNA levels of *p53* and its target genes in the *brcc3* mutant embryos (Figure 5E). Taken together, the above results suggested that depletion of Brcc3 in zebrafish embryos inhibits the repair of DSBs induced by UV irradiation, and the accumulation of DSBs activates the ATM-p53 signaling cascade and eventually leads to the increased death of the mutants.

### 2.6. Inhibition of ATM Activation Counteracts the Elevated ETO-Induced Effects in brcc3 Mutant Embryos

**Figure 6:**
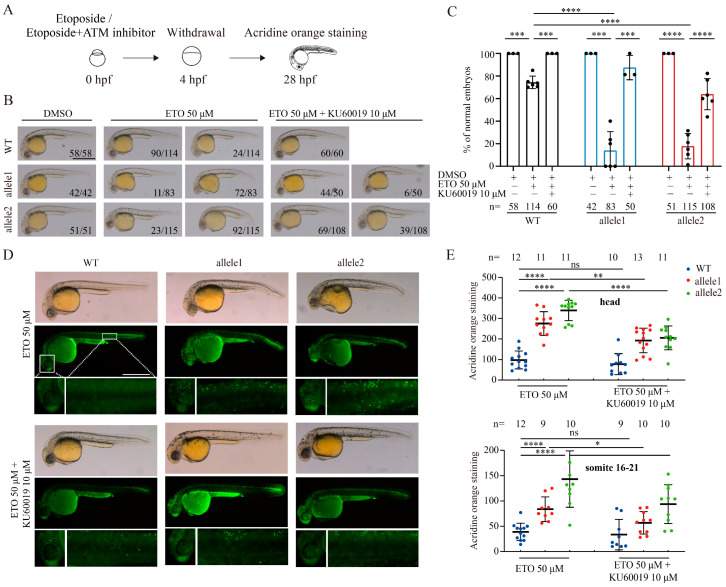
Pharmacological inhibition of ATM activation counteracts the elevated ETO-induced effects in *brcc3* mutant embryos. (**A**) Schematic diagram of WT and *brcc3* mutant embryos treated with ETO or ETO plus an ATM inhibitor. (**B**) Addition of an ATM inhibitor KU60019 rescued the phenotypic abnormalities caused by ETO treatment. The proportion of embryos with the indicated phenotypes is shown in the bottom right corner of each panel. Scale bar = 500 μm. (**C**) Quantitative results of embryos with morphological normal as shown in (**B**). The total number of embryos is shown below the column. Values are represented as means ± s.d. *** *p* < 0.001; **** *p* < 0.0001 (one-way ANOVA followed by Tukey’s post hoc test). (**D**) Acridine orange staining of WT and *brcc3* mutant embryos at 28 hpf after ETO or ETO plus KU60019 treatment. White boxes indicate local magnification. Scale bar = 500 μm. (**E**) Quantitative results from images of head or 16–21 somite regions as shown in (**D**). The total number of embryos is shown above the column. Results are from three independent replicates. Values are represented as means ± s.d. ns, not significant; * *p* < 0.05; ** *p* < 0.01; **** *p* < 0.0001 (one-way ANOVA followed by Tukey’s post hoc test).

Previous studies have shown that KU60019 inhibited the DNA damage response and apoptosis of T cells caused by ETO [33]. Therefore, the *brcc3* mutant embryos were also treated with KU60019 to evaluate whether the addition of ATM inhibitor could reverse the over enhanced ETO-induced effect after depletion of Brcc3 (Figure 6A). Indeed, treatment with KU60019 rendered the synergistically malformed degree of ETO treatment-induced phenotype and increased phenotypic percentage with normally morphological traits in *brcc3* mutant embryos (Figure 6B,C). Likewise, treatment with KU60019 also suppressed the ETO-induced synergistic elevation of apoptotic cells in the area of the head and somite 16–21 of *brcc3* mutant embryos (Figure 6D,E). These results suggest that the sensitivity to genotoxic treatment induced by the depletion of Brcc3 in zebrafish embryos can be restored by inhibiting ATM activity.

## 3. Discussion

In this study, we generated zebrafish *brcc3* knockout mutants by CRISPR/Cas9 system. We found that the *brcc3* homozygous mutants are sensitive to DSBs induced by UV radiation or by ETO treatment, although they are phenotypically normal and fertile. Since the mortality and malformation rates of the mutant with DNA damage were significantly higher than those of wild-type after UV radiation or ETO treatment, we further performed a series of rescue experiments and demonstrated that the above phenotypes were due to the failure of DSBs to be repaired in time in the mutants, which subsequently activated the ATM-p53 signaling pathway. Overall, our results showed that *brcc3*-knockout zebrafish embryos can be used as a BRCC3-deficient vertebrate model to examine its physiological role in DDR.

As mentioned earlier, BRCC3, as a component of the BRCA1-A complex, relies on its unique Lys-63 deubiquitination activity to participate in DNA damage repair through HR [8,19,35,36]. In vertebrates, the proteins involved in HR pathway are often essential for cell viability, which poses potential challenges for determining the DNA damage repair associated with HR. To overcome this shortcoming, in this study, we focused on Brcc3, which is “not crucial” for cell viability, and generated *brcc3*-deleted mutant zebrafish for physiological analysis. DNA is the primary target of genotoxic agents such as UV, which results in a pyrimidine dimer on the DNA double strand to block replication forks and cause DSBs [25]. Therefore, UV radiation was selected to further investigate the effect of Brcc3 depletion on DDR. As expected, UV radiation resulted in a dose-dependent elevation in mortality in wild-type embryos. Additionally, we found that the *brcc3* mutant embryos were more sensitive to DSBs caused by UV irradiation. In DDR, p53 is frequently phosphorylated and activated to serve as a downstream target of ATM, and involved in apoptosis induced by irreversible DNA damage [37,38]. Here, we also observed that after UV radiation, both wild-type and mutant embryos showed activation of the p53 signaling pathway. This activation is more pronounced in mutant embryos, which is consistent with their phenotypic responses to UV exposure. ETO functions as a topoisomerase II inhibitor by intercalating into replicating DNA, and thereby blocks DNA replication behind replication forks, which in turn induces DSBs and arrests the cell cycle [32,39]. Similar to UV irradiation, ETO treatment induced DSBs in zebrafish embryos, while *brcc3* mutant embryos were more sensitive than WT to ETO treatment. The DSBs caused by UV radiation or ETO treatment may be repaired through HR by BRCA-A complex, which can target the replication fork [4,7]. Thus, the hypersensitive of *brcc3* mutants in response to DNA damage likely results from insufficient DNA repair by the deficient BRCA1-A complex.

ATM is recruited to DSB, where it relies on its PIKK domain as an apical kinase to coordinate the cellular response to DSB [2]. This means that ATM can phosphorylate and activate other kinases, thereby activating multiple substrates to perform different functions, such as DNA repair, cell cycle checkpoint, modulation of signaling and metabolic pathways, and activation of cell death or senescence pathways [2,40]. Therefore, ATM can promote HR once its downstream recombinase RAD51 is activated (Figure 5A) [41]. The nuclear foci of RAD51 can be used to indicate that HR is involved in DNA repair [42]. Similarly, Ku80 can specifically mark NHEJ-dependent repair because it can recruit DNA-PK to the DNA damage site [2,43]. Another apical kinase in DDR is ATR (ATM and Rad3-related), its discovery was due to the identification of the ATM homologous gene RAD3 in fission yeast, which also contains the PIKK domain [2,44]. The function of ATM and ATR in DDR was overlapping but non-redundant [45]. ATR recognizes RPA (Replication protein A)-coated single-stranded DNA (ssDNA) with the help of its partner protein ATRIP (Figure 5A) [2,21,45,46,47]. In contrast to ATM primarily responding to DSBs, ATR is activated by a much wider range of genotoxic stresses [2]. This may explain why, when we used an ATM inhibitor KU60019 and an ATM/ATR inhibitor CGK733 to perform rescue experiments on *brcc3* mutant background, the latter showed poor efficacy. Future studies will be needed to determine the specific underlying mechanism involved in the in vivo action of the ATM inhibitor KU60019 in *brcc3* mutant embryos.

In summary, our results showed that *brcc3*-null zebrafish is more sensitive to DNA damage while it is phenotypically normal, and Brcc3 plays a vital physiological role in DNA damage response. Currently, radiotherapy and chemotherapy are key methods for treating cancer due to DNA instability in cancer cells. The increased sensitivity of *brcc3*-null zebrafish in response to DNA damage suggests that they could serve as a valuable model for screening chemotherapy drugs to expand the drug range for cancer treatments.

## 4. Materials and Methods

### 4.1. Chemicals and Reagents

All-In-One 5X RT Master Mix kit was purchased from Applied Biological Materials Inc. (Beijing, China). DIG-UTP and anti-digoxigenin-AP were purchased from Roche (Indianapolis, IN, USA). The antibody against γ-H2AX was purchased from Santa Cruz Biotechnology (1:500 for immunofluorescence, sc-517348, Santa Cruz, CA, USA). ETO (HY-13629), KU60019 (HY-12061), and CGK733 (HY-15520) were purchased from MedChemExpress (Monmouth, NJ, USA). KOD (Fx) DNA polymerase was purchased from Toyobo (Osaka, Japan). Fetal bovine serum (FBS) was purchased from PAN (Aidenbach, Germany). The qRT-PCR primers were synthesized by Sangon Bioengineering (Shanghai, China).

### 4.2. Zebrafish Strains

Wild-type zebrafish (*Danio rerio*, Tübingen strain) and p53-defective mutant zebrafish *tp53^M214K^* [48] were maintained on a 14 h light/10 h dark cycle at 28.5 °C and fed twice daily. Embryos were generated by natural cross, and reared in embryo rearing solution in an incubator at 28.5 °C. The stages of the embryos were determined according to standard methods [49]. All experimental protocols were approved by and performed according to the guidelines set by the Ethical Committee of Experimental Animal Care, Ocean University of China (Approval No. 2021035).

### 4.3. Construction of brcc3-Knockout Mutants

The *brcc3*-knockout mutants were constructed using CRISPR/Cas9 gene editing technology. The gRNA and Cas9 protein were prepared as previously described and co-injected at the one-cell stage in WT embryos [50]. The adult F0 fishes were outcrossed with WT fish to obtain the F1 generation, which was genotyped and confirmed by sequencing of targeting sites. To exclude off-target effects, the heterozygous F1 zebrafish were outcrossed with WT zebrafish for two generations.

### 4.4. UV and Pharmacological Treatments

WT or *brcc3* mutant embryos were irradiated with UV as previously described [30,51]. Briefly, zebrafish embryos were raised to 24 hpf for dechorion. The dechorionated embryos were subjected to irradiation under the UV light source with indicated exposure time. After irradiation, the embryos were raised in a dark place to the indicated stages for further analysis. The UV light source was UVC (200–280 nm) at a dose of 25 J/m^2^/s.

WT or *brcc3* mutant embryos were treated with ETO as previously described [52,53]. Briefly, two-cell stage zebrafish embryos were raised in rearing solution with ETO to 28 hpf for further analysis.

WT or *brcc3* mutant embryos were treated with an ATM inhibitor, KU60019, or an ATM/ATR inhibitor, CGK733, to block DDR as previously described [33,54]. The WT or *brcc3* mutant embryos at 22 hpf were dechorionated, pretreated with KU60019 for 2 h before UV irradiation, and then raised to the indicated stages for further analysis. One-cell stage zebrafish embryos were treated with ETO and KU60019 simultaneously for 4 h. Then, the embryos were treated with ETO only to 28 hpf for further analysis.

### 4.5. RNA Extraction and RT-PCR Analysis

Total RNA was isolated from zebrafish embryos using RNAiso Plus kit (Takara Bio, Shiga, Japan). Subsequently, 2 μg of the RNA template was reverse transcribed into complementary DNA using All-In-One 5X RT Master Mix kit (Applied Biological Materials Inc., Beijing, China), according to the manufacturer’s instructions. The cDNA was diluted 10 times to be used as the template. Quantitative PCR (qPCR) was carried out using a QuantStudio 3 Real-Time PCR Systems (Applied Biosystems, Wakefield, RI, USA). Data were collected from at least three independent experiments, and each sample was measured in duplicate. The mRNA levels of the indicated genes were calculated using 2^−ΔΔCt^ method and normalized to levels of *β-actin* [55]. The primers for qRT-PCR were listed in the Supplementary Table S1.

### 4.6. Whole-Mount In Situ Hybridization

Whole-mount in situ hybridization using DIG-labeled RNA riboprobes was performed as previously described [56,57,58].The plasmid DNA containing a partial ORF and the 3′ untranslated region of zebrafish *brcc3* was used to generate sense and antisense riboprobes. The primers of the probe were listed in the Supplementary Table S1. Images were captured using a Leica M205 microscope (Leica Microsystems, Wetzlar, Germany).

### 4.7. Immunohistochemistry

Immunocytochemistry was performed as previously described [59,60]. Embryos were fixed with 4% PFA overnight at 4 °C. After fixation, the embryos were washed in 0.2% Triton X-100 in PBS (PBST) for 10 min and blocked with 5% FBS in PBST with 1% BSA and 1% DMSO added before immunostaining for at least 1 h. The antiγ-H2AX antibody was diluted in the blocking solution and incubated at 4 °C overnight. Next, the embryos were washed in PBST, incubated with the secondary antibody Cy3, and incubated at room temperature for 2 h. After being washed with PBST, the nuclei were counterstained with DAPI. All samples were imaged using a Leica SP8 confocal or Leica M205 microscope (Leica Microsystems, Wetzlar, Germany). The γ-H2AX staining is the ratio of γ-H2AX positive signal cells to total cells with DAPI signal, and a fluorescence threshold was set to exclude background signals when analyzed by Image J (version 1.53K, NIH, Bethesda, MD, USA).

### 4.8. AO Staining

The living embryos at indicated stages were collected after ETO treatment, dechorionated, and stained with 2 μg/mL AO (Sigma-Aldrich, St Louis, MO, USA) for 30 min [61]. The embryos were then washed three times in embryo rearing solution and anesthetized with 0.08% tricaine. Images were captured using a Leica M205 microscope. AO signal was analyzed by Image J software.

### 4.9. Statistical Analysis

All experiments were repeated at least three times. All experimental data were analyzed using GraphPad Prism version 9 (GraphPad, San Diego, CA, USA). Comparisons between the two groups were performed using Student’s *t*-tests, and three or more groups were compared using one-way ANOVA followed by Tukey’s post hoc test. Values are represented as means ± s.d. and significance was defined as *p* < 0.05 or smaller *p* values.

## 5. Conclusions

DDR is crucial for maintaining genome stability, and its dysregulation can lead to the occurrence of various human diseases, including cancer, genetic disorders, and aging. To further investigate the DDR, we generated zebrafish *brcc3* knockout mutants that can respond to DNA damage. The mutant is phenotypically normal and fertile, but it is highly sensitive to DNA damage, which is induced by UV radiation or ETO treatment. Furthermore, by using an ATM inhibitor and genetic inactivation of the ATM target gene p53, successfully rescued the phenotype of the mutant induced by DNA damage. This suggests that the DNA damage in the *brcc3*-null zebrafish cannot be repaired in time, which activates the ATM-p53 signaling pathway and leads to apoptosis. These new findings provide a promising vertebrate model for real-time monitoring of DNA damage and developing new cancer therapies.

## Data Availability

All the data are provided within the article and Supporting Information Data will be shared upon request (Xiaozhi Rong, Ocean University of China, rongxiaozhi@ouc.edu.cn).

## Supplementary Materials

The supporting information can be downloaded at: https://www.mdpi.com/article/10.3390/ijms252212108/s1.
